# Development of an IMU based 2-segment foot model for an applicable medical gait analysis

**DOI:** 10.1186/s12891-024-07719-0

**Published:** 2024-07-31

**Authors:** Leandra Bauer, Maximilian Anselm Hamberger, Wolfgang Böcker, Hans Polzer, Sebastian Felix Baumbach

**Affiliations:** 1 Department of Orthopaedics and Trauma Surgery, Musculoskeletal University Center Munich (MUM), LMU University Hospital, LMU Munich, Marchioninistr. 15, 81377 Munich, Germany; 2 Experimental Orthopaedics, University Hospital Jena, Campus Eisenberg, Waldkliniken Eisenberg, Germany

**Keywords:** Gait analysis, Foot model, Biomechanical movement, Inertial measurement unit, Kinematics

## Abstract

**Background:**

The two most commonly instrumented gait analysis tools used are Optical Motion Capture systems (OMC) and Inertial Measurement Units (IMU). To date, OMC based gait analysis is considered the gold-standard. Still, it is space-, cost-, and time-intense. On the other hand IMU systems are more cost- and time effective but simulate the whole foot as a single segment. To get a more detailed model of the foot and ankle, a new 2-segment foot model using IMU was developed, comparable to the multi-segment foot models assessed by OMC.

**Research question:**

Can an IMU based 2-segment foot model be developed to provide a more detailed representation of the foot and ankle kinematics?

**Methods:**

To establish a 2-segment foot model, in addition to the previous 1-segment foot model an IMU sensor was added to the calcaneus. This allowed the differentiation between the hindfoot and forefoot kinematics. 30 healthy individuals (mean age 27 ± 7 years) were recruited to create a norm data set of a healthy cohort. Moreover, the kinematic data of the 2-segment foot model were compared to those of the traditional 1-segment foot model using statistical parametric mapping.

**Results:**

The 2-segment foot model proved to be applicable. Furthermore, it allowed for a more detailed representation of the foot and ankle joints, similar to other multi-segment foot model. The healthy cohort’s norm data set showed a homogeneous motion pattern for gait.

**Conclusion:**

The 2-segment foot model allows for an extension of IMU-based gait analysis. Futures studies must prove the reliability and validity of the 2-segment foot model in healthy and pathologic situations.

**Level of evidence:**

Level II.

## Introduction

Digital and longitudinal data acquisition will be among the most important topics in the 21st century in the fields of orthopedics. Moreover, the novel European regulation of medical devices (EU: 2017/745 and 2017/746) further increases the demand for quantitative outcome data. This constitutes a unique opportunity for instrumented gait analysis. Objectified gait parameters can be used to diagnose, assess, monitor, and predict a wide range of medical conditions [1].

The two most commonly used tools for instrumented gait analysis are Optical Motion Capture systems (OMC) and Inertial Measurement Units (IMU) [2]. To date, OMC based gait analysis is considered the gold-standard. Still, it is space-, cost-, and time-intense [3, 4]. Therefore, its applicability in the clinical routine is limited. IMU based gait analysis are a cost- and time efficient alternative to OMC [5–7]. It has been proven to have a good accuracy for the sagittal and a moderate accuracy for the frontal and transverse planes [8]. Still, currently available IMU systems are limited to the kinematics of the hip and knee. A meaningful analysis of the hind- and midfoot motion is not possible because the foot and ankle are simulated by a single sensor, i.e. a single rigid segment.

The authors are aware of two studies, who addressed these shortcomings by adding an additional IMU sensor to the foot. Rouhani et al. [4] in 2012 used the IMU system by Physilog, BioAGM, CH and more recently Okkalidis et al. [9] used an in-house build wearable system using MPU-9250, InvenSense, USA. Both studies used wire-based systems which only assessed the foot. Therefore, these systems are uncapable of performing a complete, instrumented gait analysis of the lower limb.

For the current study, the authors used the latest, wireless IMUs (Ultium Motion, Noraxon U.S.A., Inc., Scottsdale, AZ, USA) and amended the standard setup for the lower limb by an additional calcaneal sensor. Thereby, the hind- and mid-foot motion could be assessed separately, in addition to the standard kinematics of the lower limb. Such a system could be a valuable and applicable tool within the clinical routine. The aim of the current study was to develop a new IMU based 2-segment foot model and compare it to the previous used 1-segment foot model.

## Materials and methods

The herein presented study is a prospective, laboratory study on healthy individuals. The study was approved by the local ethics commission (#19–0177). All participants gave written informed consent.

### Setup of the 2-segment foot model

**Fig. 1:**
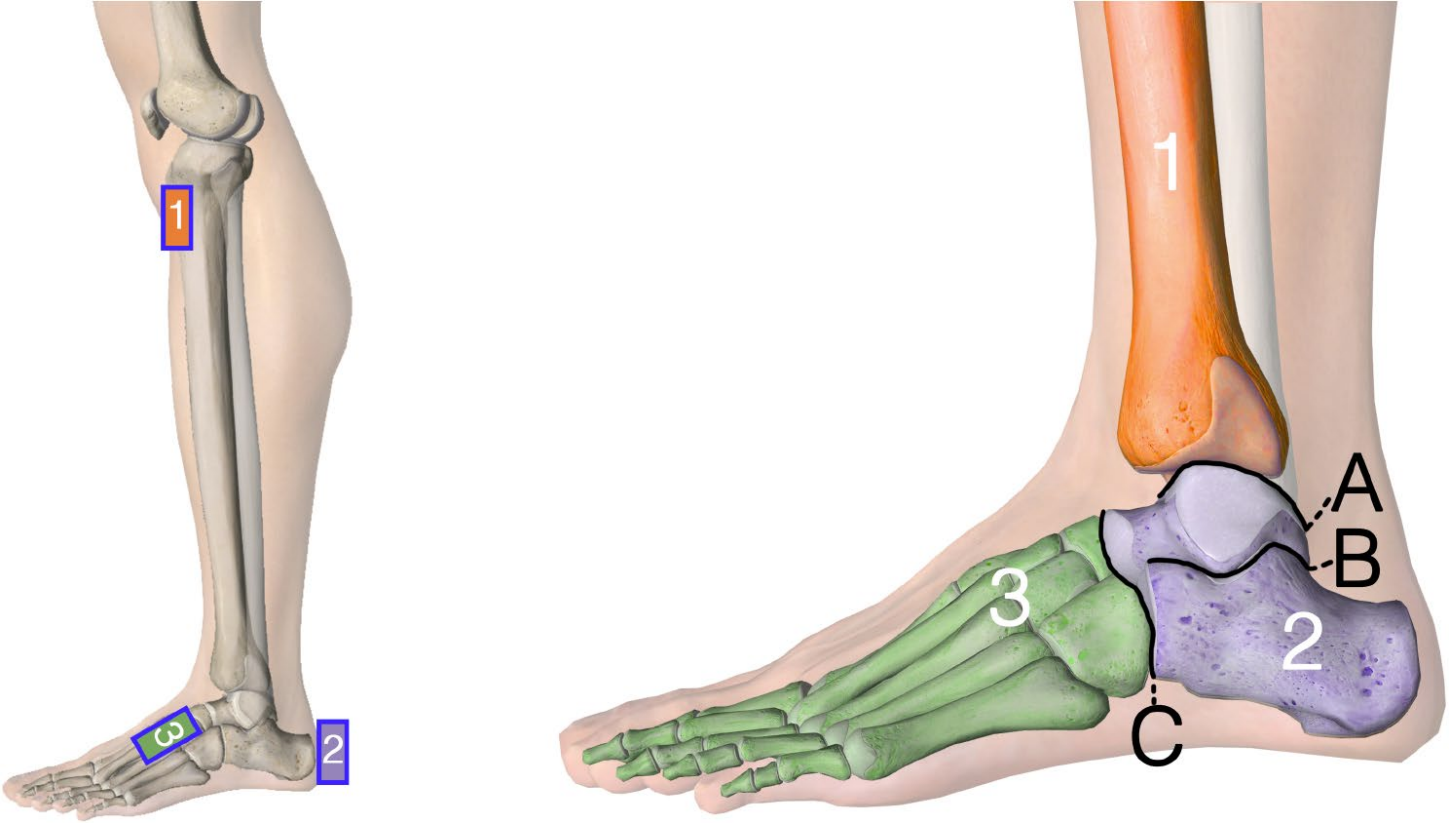
Illustration of the 2-segment foot model with the positions of the IMU sensors on tibia (1), hindfoot (2), and forefoot (3) as well as the three major joint lines: A) Ankle-, B) Subtalar-, C) Chopart-joint

To develop the new biomechanical 2-segment foot model, nine IMU sensors (Ultium Motion, Noraxon U.S.A., Inc., Scottsdale, AZ, USA) and the associated software MyoResearch (MR3.18, Noraxon U.S.A., Inc., Scottsdale, AZ, USA) were used. The seven IMU sensors were applied according to the manufacturers’ recommendation: the pelvic sensor was placed on the sacrum, the thigh sensors on the anterior distal half of the femur, the tibia sensors on the medial surface of the proximal tibia, and the standard forefoot sensors bilaterally to the dorsal midfoot [10]. The manufactures’ standard model for the lower limb represents the ankle and foot as one single rigid segment (1-segment foot model), i.e., the range of motion between the tibia (Fig. 1, #1) and the dorsum of the foot (Fig. 1, #3). To allow for a differentiation between the hind- and midfoot, an additional IMU sensor was placed vertically, centered on the posterior aspect of the calcaneus (2-segment foot model). The sensors at the pelvis, dorsum of the feet, and the calcaneus were secured using body adhesive strips. For additional support, the subjects’ socks were pulled over the feet. The sensors on the pelvis, thigh and tibia were fixed with straps. The final setup of the 2-segment foot model is illustrated in Fig. 1.

The additional calcaneal sensor was added to the biomechanical model in the associated software (MyoResearch MR3). This allowed to amend the standard joint angle calculations by the tibio-calcaneal- (ankle joint) and calcaneal-midfoot (subtalar/chopart joint) angles. Next, the angle calculations in x-, y- and z-plane (equals transverse, sagittal, frontal plane) between the tibia and hindfoot, as well as the hindfoot and forefoot were implemented by using the biomechanical model of the software. This setup allowed an approximation to the three major joint axes of the hindfoot, and therefore an analysis similar to other multi-segment foot models (Table 1).

**Table 1:** Analysis of the 2-segment foot model in approximation to the oxford foot model

	Sagittal plane	Frontal plane	Transversal plane
Hindfoot (#2) - Tibia (#1)	Ankle (Plantar-/Dorsiflexion)	Subtalar (Varus/Valgus)	Subtalar (Internal Rotation/ External Rotation)
Forefoot (#3) – Hindfoot (#2)	Chopart joint (Plantar-/Dorsiflexion)	Chopart joint (Supination/Pronation)	Chopart joint (Forefoot Ab-/ Adduction)
Standard Model Tibia (#1) – Forefoot (#3)	Ankle & Chopart joint (Plantar-/Dorsiflexion)	Ankle & Subtalar & Chopart (Supination/Pronation)	Tibia - Forefoot (Ab-/Adduction)

### Study participants

Healthy subjects were recruited to record reference data to establish a norm data set. Inclusion criteria were age > 18 and < 60 years, as well as no relevant injuries or previous diseases of the lower extremities and comorbidities.

### Gait analysis

All subjects performed a standardized gait analysis protocol. The gait analysis was performed on a treadmill (pluto med, h/p/cosmos sports & medical GmbH, Nussdorf-Traunstein, Germany) with an integrated pressure plate (Zebris Medical GmbH, Isny, Germany). The data recording occurred time-synchronized in the software MyoResearch.

Nine IMU sensors were placed according to the above outlined setup. The calibration of the sensors was done with a dynamic walking calibration: First a reference pose measurement was conducted with the participants standing straight. Next a walking calibration was performed. The defined movement of the walking calibration can be used to correct the course orientation of the sensors, utilising the knowledge of the segment movement during walking. The sensor drift is further corrected by the initial and final reference pose. Finally, the actual gait analysis was conducted. The participants started with a familiarization period on the treadmill for 2 min. All participants walked at the same speed of 4 km/h. The actual recording of the data started automatically for a duration of 30 s immediately following the familiarization period.

### Data collection and processing

The time required to perform the IMU based instrumented gait analysis, the foot and ankle kinematics, their visualization, and the individual range of motions (ROM) were evaluated. The values obtained for the 2-segment foot model were compared to the standard values provided by the 1-segment foot model.

The duration of the gait analysis was obtained from the data acquisition software. The software records the starting time of the walking calibration and the gait analysis. This allowed to calculate the time for the actual gait analysis. The time needed for sensor placement, data backup, and sensor removal was not recorded and therefore could not be assessed.

All IMU data were received wirelessly, were synchronised via a receiver, and forwarded to the PC. The recording frequency was 200 Hz. An object sensor was added to the MR3 software for the additional sensor at the heel. The kinematics of the segments in three planes (sagittal, frontal, transverse) for the ankle and subtalar/chopart joints were calculated in the MR3 software in accordance with the recommendations of the International Society of Biomechanics (ISB) as a cardanic rotation sequence [11]. Further data processing was done in Matlab (The MathWorks Inc., Natick, MA, USA). The data were interpolated to 100% gait cycle in 1% increments. Both feet of each subject were used to calculate the norm values for a healthy cohort. Next mean values and standard deviations were calculated for the whole gait cycle.

The visualization of the data was divided into the Tibia/Forefoot, Hindfoot/Forefoot, and Tibia/Hindfoot kinematics for the sagittal, frontal, and transversal planes using the mean values and a standard deviation.

In addition, the total ROM of each joint was calculated from the minimum and maximum values. These were used to compare them with values with multi-segment foot models measured by OMC from the literature. To determine the differences between the 1-segment and the 2-segment foot model, the kinematic curves were compared using the statistical parametric mapping (SPM) with a paired t-test and an alpha level of 0.05 (Copyright (C) 2021 Todd Pataky, available at spm1d.org) [12].

## Results

### Development of the new 2-segment foot model

The attachment of the additional sensor to the calcaneus proved to be stable. In none of the assessed cases, the sensors had to be adjusted or re-placed. The additional foot segments were successfully implemented in the software, allowing for automated calculation of all joint angles. By exporting the data from the software, a 9-field graphic with the kinematics of the foot/ankle joint in all three planes (sagittal, frontal, transverse) could be displayed using a custom Matlab (MathWorks, Inc., Natick, MA, USA) script. This graphic combines the angles from the old 1-segment foot model and the new 2-segment foot model.

### Duration of data assessment

The average data acquisition for the calibration, i.e. from starting the walking calibration to the start of the familiarization period, took on average 5 ± 4 min. The time for familiarization on the treadmill and the actual recoding period was 2.5 min, which must be added to the 5 ± 4 min of calibration time. Therefore, the total time of data acquisition was 7.5 ± 4 min.

### Norm data

30 healthy individuals (14 female, 16 male) were recruited with a mean age of 27 ± 7 years, mean body weight of 69 ± 12 kg and a mean body height of 176 ± 10 cm.

**Fig. 2:**
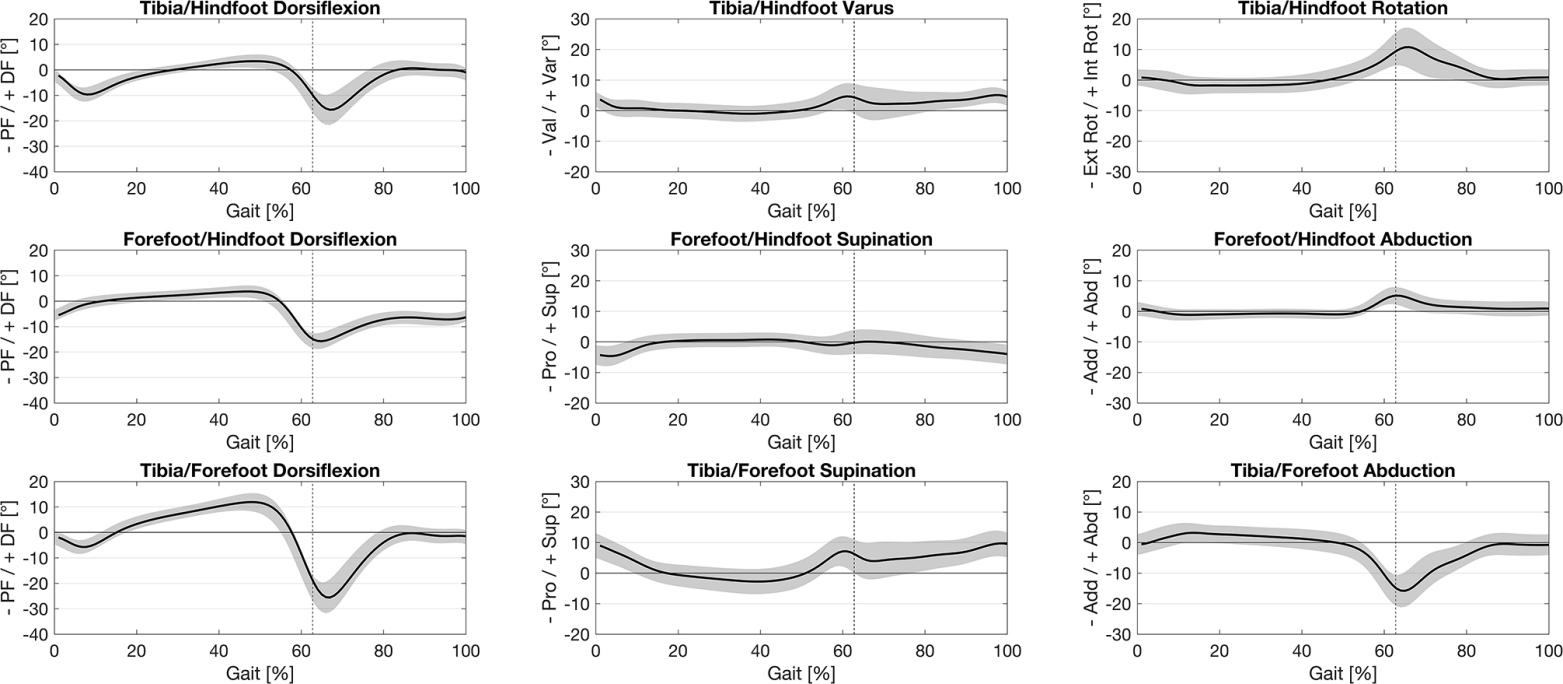
Norm data for the 2-segment foot model with mean (± standard deviation) of 100% gait cycle for Tibia/Hindfoot, Forefoot/Hindfoot, Tibia/Forefoot in sagittal, frontal and transverse plane. PF – Plantarflexion, DF – Dorsiflexion, Pro – Pronation, Sup - Supination, Add – Adduction; Abd – Abduction

Tibia/Forefoot dorsiflexion showed the highest ROM over the full gait cycle compared to Tibia/Hindfoot and Forefoot/Hindfoot dorsiflexion (Table 2). The analysis of the Tibia/Hindfoot movement in frontal plane corresponds to the varus/valgus movement of the hindfoot. At the end of the stance phase, there was a clear varus movement of the hindfoot, which decreased with the middle swing phase (Fig. 2). The ROM of Tibia/Hindfoot was 20.2° ± 4.7° in the sagittal plane and 8.9° ± 3.2° in the frontal plane. For Forefoot/Hindfoot, a ROM of 20.2° ± 3.0° in the sagittal plane and 7.4° ± 2.3° in the frontal plane was measured (Table 2).

**Table 2:** Comparison of range of motion values between the 2-segment foot model and previously published data from OMC based studies on healthy individuals. Values are given as mean (± standard deviation, when available)

	T/HF Sagittal	FF/HF Sagittal	T/HF Frontal	FF/HF Frontal
**Bauer et al. (2023)** (IMU based 2-segment foot model)	20.2° ± 4.7°	20.2° ± 3.0°	8.9° ± 3.2°	7.4° ± 2.3°
**Schallig et al. (2020)** (OMC based OFM)	27.4° ± 3.3°	12.5° ± 3.5°	10.0° ± 1.9°	8.6° ± 2.2°
**Wang et al. (2010)** (OMC based OFM)	21.9° ± 4.9°	15.4° ± 3.6°	10.9°	6.6°
**Levinger et al. (2010)** (OMC based OFM)	21.2°	14.4°	13.2°	7.3°

T/HF: Tibia/Hindfoot, FF/HF: Forefoot/Hindfoot; Frontal: Frontal plane; Sagittal: Sagittal plane

### Comparison 1-segment to 2-segment foot model

**Fig. 3:**
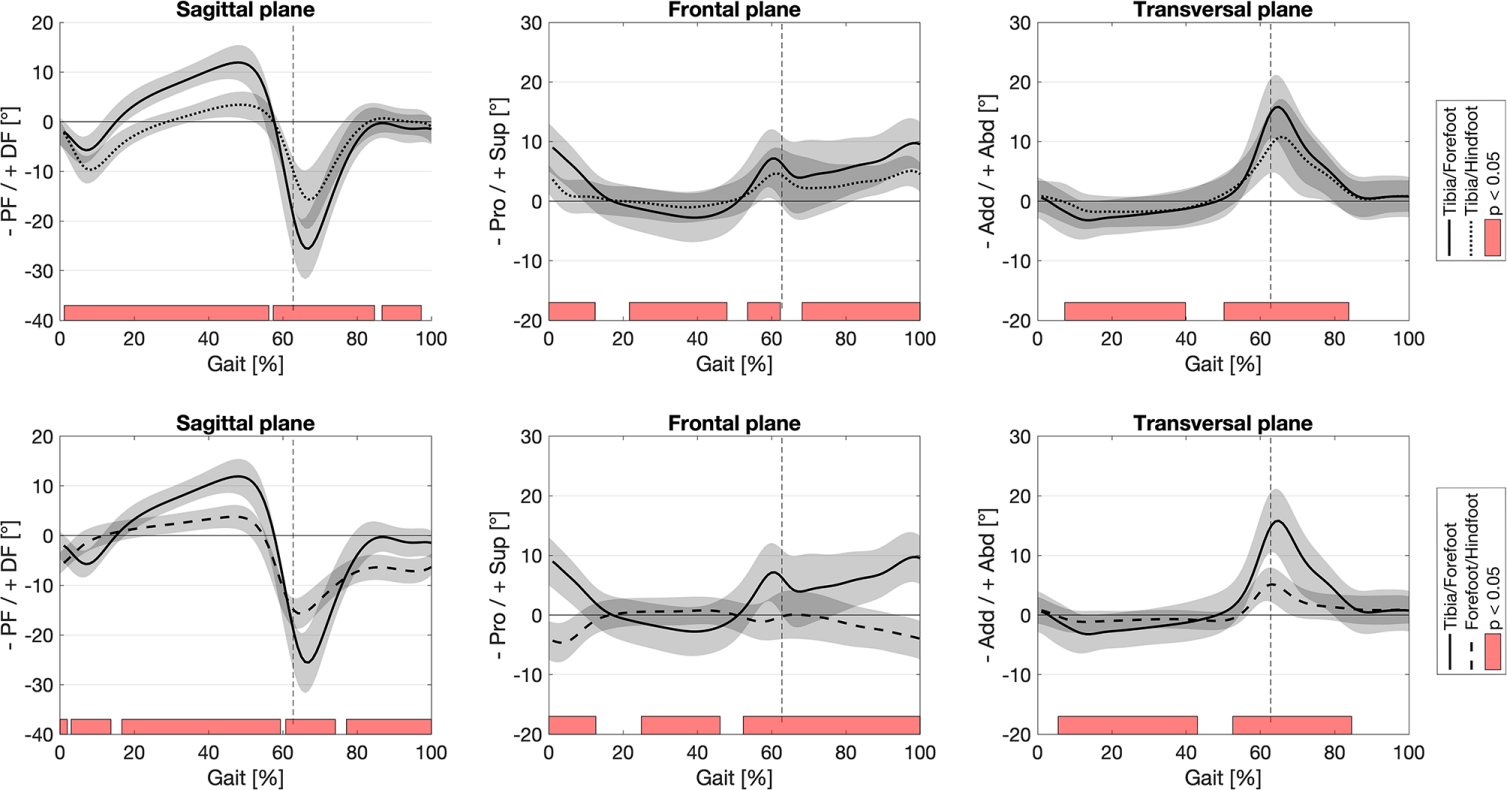
Illustration comparing the six kinematic curves derived from the 2-segment foot model (Tibia/Hindfoot, Forefoot/Hindfoot) to the corresponding curves of the traditional 1-segment foot model (Tibia/Forefoot). PF – Plantarflexion, DF – Dorsiflexion, Pro – Pronation, Sup – Supination, Add – Adduction; Abd – Abduction; Red bars indicate areas of significant differences based on the statistic parametric mapping

Comparison of the six kinematic curves derived from the 2-segment foot model (Tibia/Hindfoot, Forefoot/Hindfoot) with the corresponding curves from the traditional 1-segment foot model (Tibia/Forefoot) revealed significant differences throughout the whole gait cycle (Fig. 3). These differences included the shape and amplitude of the individual kinematic curves, indicating a more detailed representation of the foot and hindfoot kinematics for the 2-segment foot model. The mean difference ± 1 SD [minimum; maximum] between the two models for the three planes were for Tibia/Forefoot vs. Tibia/Hindfoot: sagittal plane 5.0 ± 2.9 [-10.3; 8.5], frontal plane 2.3 ± 1.4 [-1.9; 5.8], transverse plane 1.1 ± 1.3 [-1.8; 5.5]; for Tibia/Forefoot vs. Forefoot/Hindfoot: sagittal plane 5.0 ± 2.5 [-10.6; 8.2], frontal plane 5.6 ± 3.8 [-3.5; 13.6], transverse plane 2.5 ± 3.0 [-2.1; 11.0].

## Discussion

The main result of this study was the successful development of an IMU-based 2-segment foot model. The model proved to be a significant evolution of the currently available commercial 1-segment foot model. It showed a homogeneous gait pattern for the hind- and midfoot and allowed a more detailed representation of the foot and ankle kinematics.

Next to establishing the methodology of a 2-segment foot model, the authors presented a norm value data for a healthy cohort set based on 30 individuals. Table 2 compares the herein established norm values, i.e. maximum ROM, to previous studies using a multi-segment foot model, i.e. Oxford Foot Model (OFM), measured by an OMC system [13–15]. Overall, there is a moderate variation, between the different OMC studies similar to those obtained from the 2-segment foot model. The values obtained from the IMU based 2-segment foot model were in the range of the OMC based values. Still, the IMU based model resulted in slightly lower ROM values for the sagittal ankle movement (Table 2: T/HF Sagittal) and in higher values for the sagittal Chopart movement (Table 2: FF/HF Sagittal). In the frontal plane, the 2-segment foot model ROM values were in line with the published values from a multi-segment foot model by an OMC setup.

The two studies [4, 9] that previously developed a multi-segment foot model with IMUs have the main disadvantage of not being able to perform a full gait analysis of the lower limbs with this setup. A in-house developed system was used in parts. In addition, wire-based IMU sensors were used here, which limits the practicality in the application. The IMU system used here in the setup and the newly developed 2-segment foot model can be implemented in commercially available software and is shown to be easily applicable due to wireless IMU sensors. In addition, gait analysis of the complete lower extremity (including knee and hip) is possible.

Comparing gait analysis in specific related to the foot and ankle joint amongst each other (IMU and OMC) is limited. First, the gait analysis can either be collected on an instrumented treadmill or during overground walking. Yang and King (2016) showed that the gait parameters for overground walking and treadmill walking differ. On a treadmill, participants tended to adopt a more cautious gait, resulting, amongst others, in a slower walking speed with shorter stride length, less backward inclined trunk, shortened single stance phase, and prolonged double stance phase [16]. The different speeds (treadmill consistent vs. overground individual, heterogeneous) may also have an impact on the overall ROM of the foot [17]. Second, the technology used, i.e. OMC or IMUs, has an impact on the data acquired. In OMC-based gait analysis, subjects conduct overground walking and usually must hit specific force plate(s). Several repetitions are performed, but only the few steps on the force plate(s) per repetition are included in the evaluation. In gait analysis using an instrumented treadmill, significantly more steps are analyzed. For the current study, all steps within 30 s at a constant speed of 4 km/h were recorded. Consequently, OMC gait analysis is based on only the average of only a few steps and instrumented treadmill analysis on, in the herein presented study, an average of 25 steps. This might have an impact on the values (mean ± SD) calculated. Finally, OMC-based gait analysis allows to define anatomical axis and segments by precise placement of multiple markers on anatomical landmarks. IMU-based gait analysis facilitates fewer larger sensors. In order to acquire consistent kinematic measurements, patients initially perform a reference pose, which is used to define neutral axis between the markers. This is less a limitation for long bones like the femur or tibia, but a source of error for the calcaneal sensor. Therefore, the physiological hindfoot valgus during easy stance is set to zero degrees. Although this likely does not affect the overall detected ROM or shape of the kinematic curve, it might result in a shift of the curve. All of these factors must be anticipated when comparing different gait analysis modalities to each other. Therefore, the ROM results from our new model appear to be comparable to those from other multi-segment foot models measured by OMC.

As mentioned previously, one major reason for the authors to focus on an IMU-based instrumented gait analysis were its applicability in the clinical routine. Compared to an OMC system, IMU systems are more time efficient, have lower acquisition costs, require less space, and the sensors can be used mobile and are not tied to laboratory conditions. A major advantage over the OMC systems is the time required to conduct the measurements and analysis. As outlined in the current paper, data acquisition in IMU based gait analysis requires 7.5 ± 4 min. Further added must be the time for preparation and attachment of the sensors (~ 5 min) and time for dismounting the sensors and saving / verification of the data (~ 5 min). Instantly from the system, a comprehensive report can be printed out and handed to the patients. This adds to a total of 17.5 min. Due to the more complex mounting of the markers, data acquisition, and data analysis, an OMC-based gait analysis requires 1 to 1.5 h for a single patient. Consequently, IMU-based gait analysis can be performed in a regular-sized room alongside a regular outpatient visit. Moreover, due to its mobility, IMU based gait analysis can also be performed on the ward with frail or post-surgical patients, which do not tolerate a strenuous OMC based gait analysis.

It should be noted that the current IMU-based model is only based on 2 segments and, unlike the OFM, does not take the hallux segment into account. Unfortunately, due to the sensor size, it has not yet been possible to find an approach that makes it possible to include the hallux segment even with an IMU. Therefore, no statement can be made about the kinematics of the flexion of the hallux segment in a gait analysis with the currently described model.

Traditional IMU-based gait analysis has been used to assess knee- and hip kinematics. With the addition of an additional sensor on the calcaneus, it can now also be used to assess meaningful ankle kinematics. The 2-segment foot model allows to differentiate between the forefoot and hindfoot, as well as ankle and subtalar joint. This differentiation is essential for a meaningful gait analysis in more complex foot and ankle deformities, such as osteoarthritis, pes planovalgus or cavus feet. Compared to OMC systems, the IMU-based gait analysis has the further advantage of being appliable outside a specific gait laboratory. Therefore in-field gait kinematics can be assessed.

## Conclusion

The herein developed and applied IMU-based instrumented gait analysis facilitating a 2-segment foot model proved applicable, time-efficient, and a clinical meaningful amendment to the previous used IMU based 1-segment foot model. The 2-segment foot model thus extends its applicability to everyday clinical practice including foot-specific pathological gait patterns. A subdivision into ankle and subtalar/chopart joint is reasonably possible and showed good results in the whole ROM compared to previous OMC based studies. Still OMC- and IMU-based instrumented gait analysis have specific advantages and disadvantages. Futures studies must prove the reliability and validity of the 2-segment foot model and depict the specific use cases for either methodology, OMC or IMU.

## Data Availability

The datasets generated and/or analysed during the current study are available from the corresponding author on reasonable request.
